# Correlation between gastrointestinal morphological changes, enteric microbiota, and changes in live weight in dairy calves

**DOI:** 10.3168/jdsc.2024-0620

**Published:** 2024-10-30

**Authors:** John Alawneh, Timothy Olchowy, Mohammad Mahmudul Hassan, Rachel Allavena, Martin Soust, Rafat Al Jassim

**Affiliations:** 1 School of Veterinary Science, The University of Queensland, Gatton, Queensland 4343, Australia; 2 Faculty of Veterinary Medicine, University of Calgary, Calgary, Alberta T3R 1J3, Canada; 3 Martin Soust & Co. Pty. Ltd. and GR International Pty. Ltd., Melbourne 3004, Australia; 4 Queensland Alliance for Agriculture and Food Innovation, St Lucia, Queensland 4072, Australia

## Abstract

This study aimed to quantify the association between fecal microbiota biomarkers, gastrointestinal tract morphology, and ADG of dairy calves from birth until weaning in response to feeding a direct-fed microbial (DFM) supplement as part of their milk diet. We randomly assigned 44 newborn Holstein-Friesian calves to treatment (TRT) and control (CON) groups. The TRT group calves received a once-daily dose of *Lacticaseibacillus*- and *Lentilactobacillus*-based DFM liquid formulation. Four genera, *Prevotella*7, *Succiniclasticum, Terrisporobacter*, and *Carnobacterium*, were enriched and identified as biomarkers of low ADG. A total of 14 bacterial taxa were associated with measured gastrointestinal histopathology variables in TRT and CON groups. Although this study lists several bacterial taxa that have known roles in fermentation and nutrient metabolism vital for rumen function, their specific contributions to gastrointestinal development and weight gain remain to be fully understood. Our findings support a strategic approach to probiotic use in heifers to boost health and productivity.

## Highlights

• Calves supplemented with DFM showed enhanced GIT morphology.
• The TRT group exhibited increased weaning weights.
• Four bacterial genera were associated with low ADG.
• Specific bacteria were correlated with GIT development

## Graphical Abstract

**Figure:**
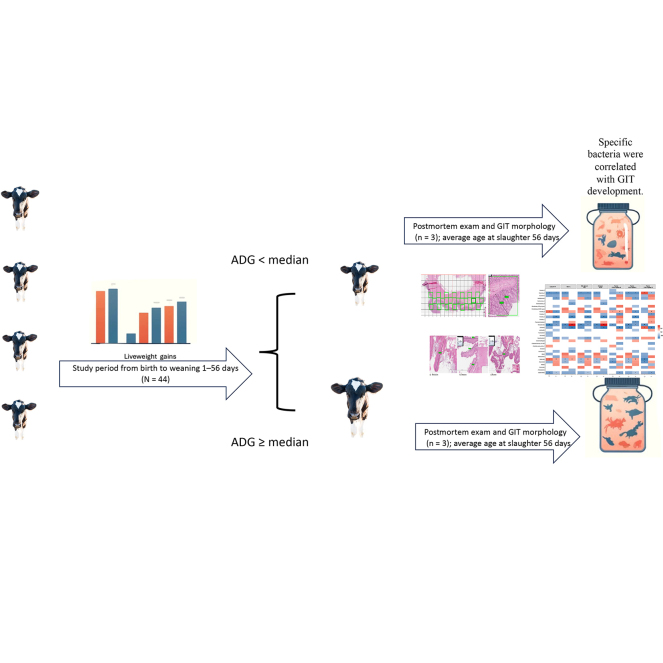
**Summary:** Above: Illustration of the impact of a lactobacilli-based direct-fed microbial (DFM) supplement on dairy calves. The centerpiece displays 2 calves, highlighting notable differences in live weight between the treatment (TRT; n = 22) and control (CON; n = 22) groups. This difference was associated with a more developed gastrointestinal tract (GIT) and the microbial community. This study demonstrates the potential of targeted microbial interventions in improving dairy calf performance and overall health, suggesting a promising avenue for enhancing livestock productivity through microbiota intervention.

Solid feed intake triggers ruminal fermentative processes in calves and enriches the indigenous microbiota, with a significant shift during weaning due to alterations in ration composition that affect ruminal and intestinal microbiomes (Schofield et al., 2018; Du et al., 2023a). The concept of using direct-fed microbials (**DFM**) becomes significant within this context (Barreto et al., 2021). An effective DFM should be tailored to support the proliferation of the indigenous microbiota and beneficially inhabit the calf's gastrointestinal tract (**GIT**; Virgínio Júnior and Bittar, 2021; Du et al., 2023a). Studies by Arshad et al. (2021) and Du et al. (2023b) have emphasized the importance of strategic microbiome management. Novak et al., (2012) evaluated the probiotic effects of *Bacillus amyloliquefaciens* on the intestinal microbiota and growth performance in dairy calves. The authors reported significant improvements in growth metrics linked to gut microbiota modulation. These findings suggest that targeted manipulation of the gut microbiota from an early age not only mitigates health issues such as diarrhea (Wang et al., 2023), but also establishes a healthier growth trajectory.

Research in dairy calves has demonstrated that probiotics can modify gut bacterial populations, potentially leading to improved growth metrics (Renaud et al., 2019; Nalla et al., 2022; Alawneh et al., 2024). Supplementation with lactic acid–based probiotics increases weaning weights, although ADG and feed efficiency remain unchanged (Wang et al., 2023). This selective benefit suggests that although probiotics may not universally enhance all growth parameters, they may still offer significant benefits by increasing overall live weight at critical periods. The identification of specific bacterial genera associated with lower ADG, such as *Prevotella*, underscores the complex role of the gut microbiome in growth, suggesting that a targeted approach to probiotic applications could pave the way for optimal precision microbiome management in agriculture (Várhidi et al., 2022). In a recent study, the authors reported GIT morphological changes and growth of dairy calves receiving a daily dose of a DFM liquid formulation containing *Lacticaseibacillus paracasei, Lentilactobacillus buchneri*, and *Lentilactobacillus casei* as part of their milk diet (Alawneh et al., 2024). Considering this observation, we hypothesis that the enteric microbiota community could have changed in response to DFM treatment. This study aimed to quantify the association between fecal microbiota biomarkers, GIT morphology, and ADG of dairy calves from birth until weaning.

A more comprehensive description of the study materials and methods has been previously reported (Hewitt et al., 2020; Alawneh et al., 2024). In summary, this was a longitudinal study conducted between June and October 2018 at the University of Queensland–Gatton Commercial Dairy, Australia (animal ethics approval no. SVS/128/18). Forty-four Holstein-Friesian calves were randomly chosen and housed individually after separation from their dams at birth. They received 2 L of colostrum within 8 to 12 h of birth and were provided with ad libitum water, calf-starter pellets, and pasture hay. A milk replacer (125 g powder/L; Norcovite, Norco, Queensland, Australia) was offered. A simple randomization without replacement technique was used to randomly assign treatment (control [**CON**] or treatment [**TRT**]) to the enrolled calves. Treatments were administered by mixing 1 mL of the liquid DFM supplement (containing a minimum of 10^9^ cfu/mL each of *Lacticaseibacillus paracasei, Lentilactobacillus buchneri*, and *Lentilactobacillus casei*; Mylo, Terragen Biotech, Queensland, Australia), or placebo with calf's milk replacer meal. Calves were weighed, and fresh fecal samples were collected (on d 0, 14, 28, 42, and 56) by stimulating the rectum, and stored at −80°C. A full description of DNA extraction and 16S rRNA gene amplicon sequencing methodology has been previously reported (Alawneh et al., 2024). After weaning, 3 male Holstein-Friesian calves each from the CON and TRT groups were slaughtered and submitted for postmortem examination. A gross necropsy examination was performed to evaluate sections of the gastrointestinal tract, and tissue samples were collected from forestomach, abomasum, duodenum, jejunum, ileum, cecum, and colon for histological analysis. Histological measurements included papilla and villus parameters obtained using systematic random sampling methods. Linear measurements were made using fractionator sampling. Surface area measurements were acquired using microscopy and software analysis. This comprehensive approach provided insights into postweaning gastrointestinal morphology changes in calves.

All analyses were conducted in R version 4.3.3 (R Development Core Team, 2024). A mixed-effects linear model was fitted to the data to estimate calf live weight as a function of calf age (days), breed, and sex. The model was fitted with the calf as a random intercept and age as a random slope. All analyses were conducted using nlme and lme4 (Bates and Maechler, 2010) statistical packages. The continuous variable ADG was categorized into low and high based on the median value (low ADG = calf ADG < median ADG; high ADG = calf ADG ≥ median ADG). Differential amplicon sequencing variants (**ASV**) abundance at the genus level was also compared between groups. A comparison of the differential ASV abundance among low and high ADG groups was carried out using differential gene expression analysis in DESeq2 (Love et al., 2014) using experimental groups (CON vs. TRT) as a covariate and the Benjamini–Hochberg adjustment for multiple tests (Douglas et al., 2020). Nonparametric Spearman rank correlation coefficient (ρ) was carried out to test the relationship between the rumen and intestinal histopathology measurements and the bacterial communities present in the fecal samples collected over the study period. For this analysis, the epidemiological unit was the GIT histological sample. A subset of the microbial abundance data related to calves selected for postmortem examination (n = 6) was extracted from the dataset and used to produce the correlation matrix. The resulting correlation matrix was visualized in a heatmap format generated by the corrplot package (Wei and Simko, 2021) in R.

Average daily gain, total feed intake, and average feed efficiency were not statistically different between the groups. Median ADG was 0.52 kg (interquartile range [**IQR**] = 0.24) for the CON group and 0.54 kg (IQR = 0.11). Four genera, *Prevotella7, Succiniclasticum, Terrisporobacter*, and *Carnobacterium*, were enriched and identified as biomarkers of low ADG (*P* < 0.001; Figure 1). Only genera detected in fecal microbiota with a relative abundance of at least 0.1% of the bacterial community in at least one calf were compared with the histopathology measurement variables (Figure 2). We found 14 ASV to be associated with measured variables in CON and TRT gastrointestinal histopathology measurements. Of those, 3 ASV were unclassified at the genus level and are presented at the phylum level (Figure 2). *Alloprevotella* was associated with CON calves and strongly correlated (ρ = −0.61; *P* = 0.02) with the villi lengths in the ileum, jejunum, and omasum, and negatively correlated (*P* = 0.02) with rumen measurements. On average, except for rumen ventral sac papillae width in the CON group (ρ = −0.68; *P* = 0.001), *Bacteroides* was positively correlated (ρ > 0.53; *P* ≤ 0.05) with all measured variables in both the CON and TRT groups. *Firmicutes, Subdoligranulum, Butyricicoccus*, and *Ruminiclostridium9* were strongly negatively associated with ileum villi length and width of omasal villi (ρ = −0.57, *P* = 0.03; ρ = 0.53, *P* = 0.04; ρ = −0.55, *P* = 0.03) in the TRT group. *Faecalibacterium* was negatively correlated with cecal, jejunal, and ruminal ventral sac papillae length (ρ = −0.52; *P* = 0.05). *Prevotellaceae Prevotella7* was also negatively associated with the rumen blind sac papillae width in the TRT calf group (ρ = −0.58; *P* = 0.02). The Parabacteroides correlations with histopathology measurements varied between the CON and TRT groups. A strong negative correlation was observed for ileum villus length (ρ = −0.67; *P* = 0.01) and omasum villi width (ρ = −0.67; *P* = 0.01) in the CON group compared with strong positive correlations in TRT (ρ = 0.74; *P* < 0.01 and 0.75; *P* < 0.01, respectively). A similar pattern was also observed for *Odoribacter* and rumen ventral sac papillae width in the CON group. *Prevotella2* was strongly negatively correlated (*P* < 0.01) with papillae length in the cecum, ileum, jejunum, omasum, and the ventral sac of the rumen in the TRT calves. *Prevotella9* was positively correlated with all measured variables in both groups, except for cecum width (ρ = −0.61; *P* = 0.02), jejunum villi length (ρ = −0.60; *P* = 0.02), and the ventral sac of the rumen (ρ = −0.61; *P* = 0.01) in the TRT calves. A similar pattern was observed for *Treponema2*.

**Figure 1:**
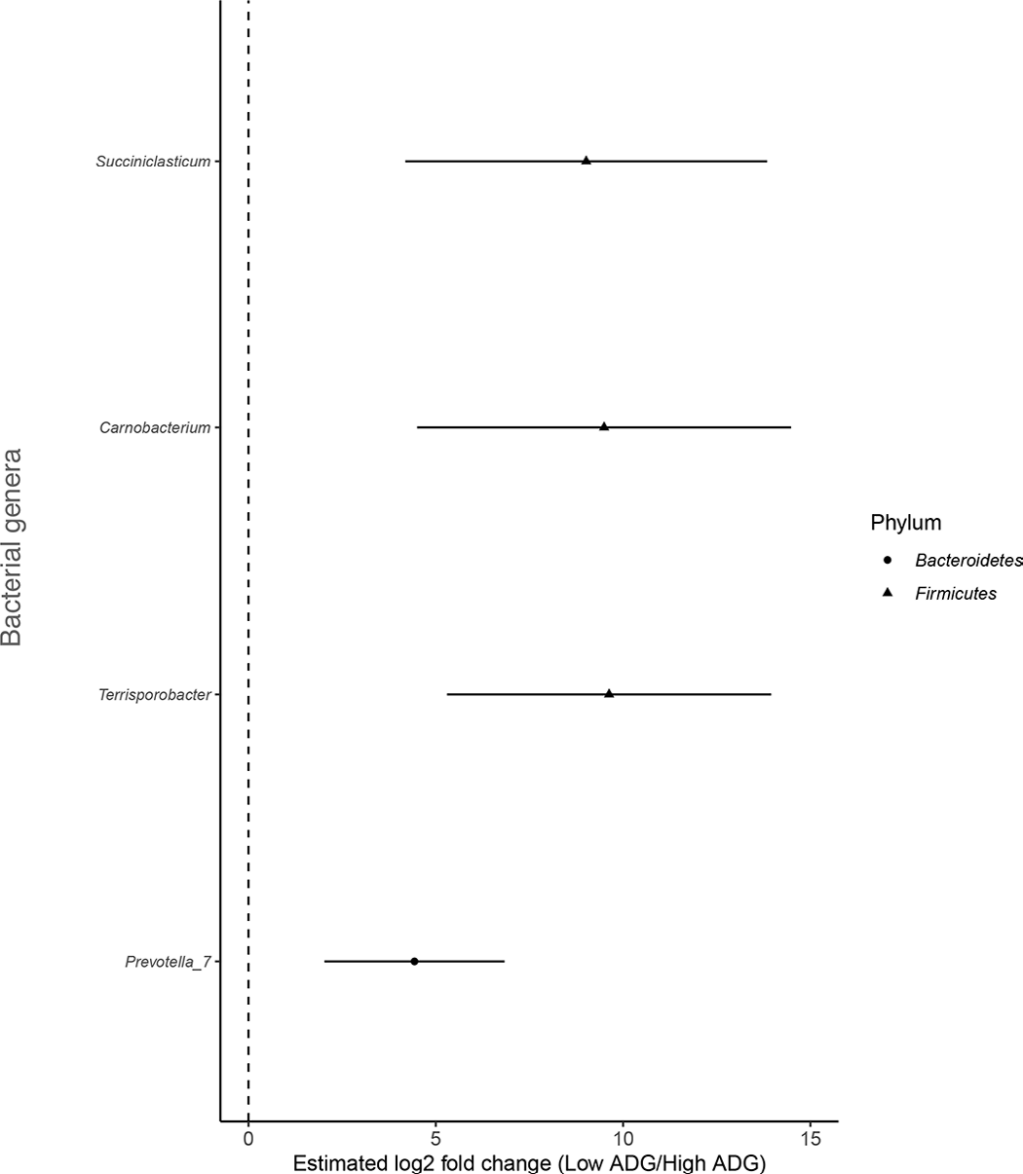
Significant (*P* ≤ 0.01) log_2_ fold differences in abundance of bacterial families between low versus high ADG.

**Figure 2:**
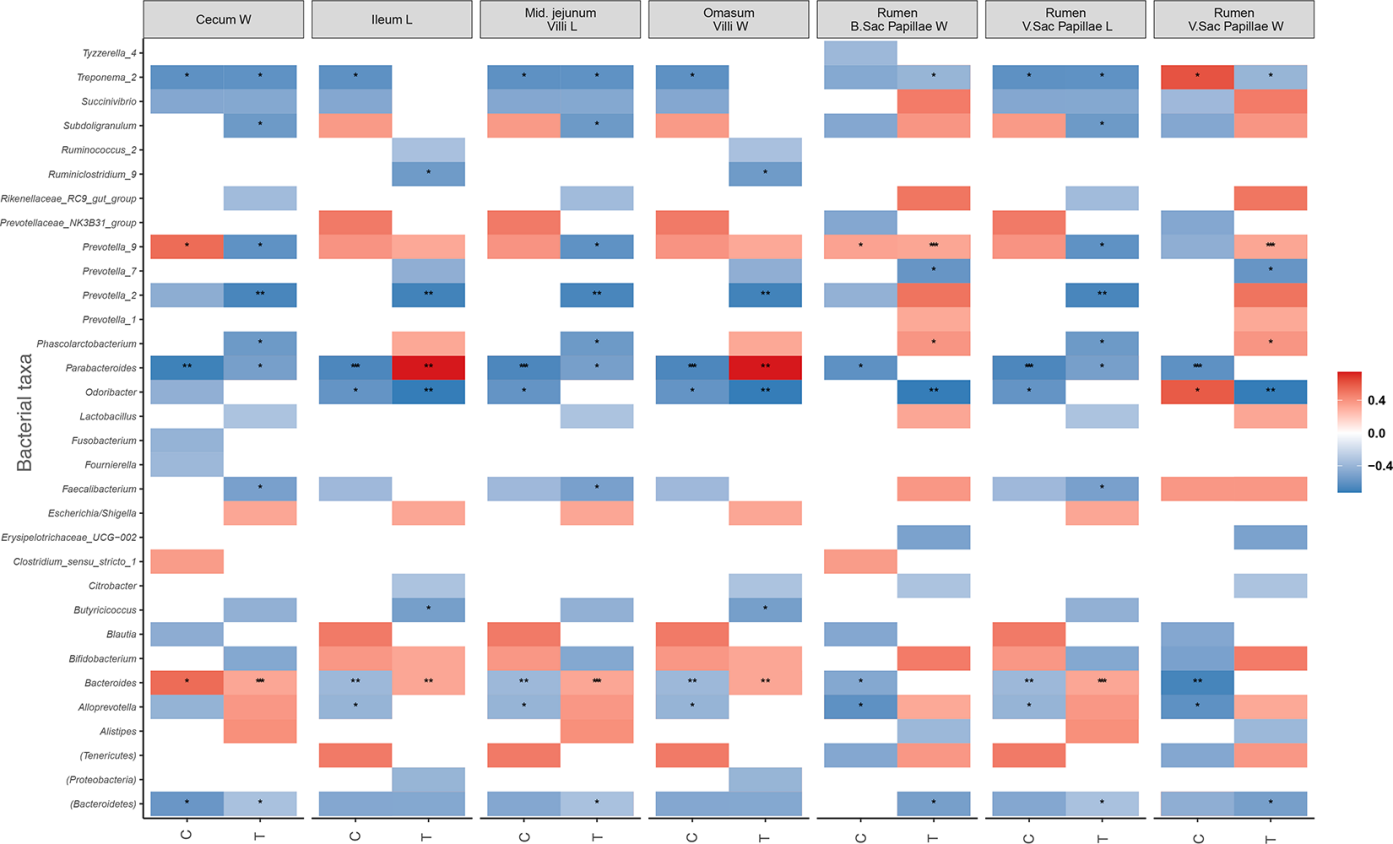
Spearman nonparametric rank correlations between rumen and intestinal histopathology measurement variables and relative taxa abundance in the control (C) and treatment (T) groups. All correlations presented were statistically significant (*P < 0.05, **P < 0.01, ***P < 0.001). The scale colors denote whether the correlation is positive (closer to 1, red) or negative (closer to −1, blue). *P*-values were adjusted for multiple comparisons. B = blind; L = length; Mid. = middle; W = width; V = ventral.

The supplementation of DFM in dairy calf diets has shown promising results. A randomized clinical study in Ontario, Canada, reported that the administration of a multispecies probiotic and yeast bolus to calves had a positive influence on health outcomes (Renaud et al., 2019). Similar findings were reported elsewhere (Jatkauskas and Vrotniakiene, 2014; Várhidi et al., 2022). The impact on ADG and feed efficiency remains inconsistent, suggesting that variations in outcomes may be affected by probiotic strain, baseline microbiota, and environmental conditions (Markowiak and Ślizewska, 2018). In this study, 4 bacterial genera were associated with lower ADG. This finding adds to the complex narrative of the role of the gut microbiota in animal health and necessitates a tailored approach to the use of probiotics in animal production if maximal beneficial effects are to be achieved (Nalla et al., 2022).

Several studies have explored the complex relationship between specific bacterial genera and live weight gain in cattle. *Prevotella*, a predominant genus in the rumen microbiota, has garnered significant attention due to its involvement in fiber degradation and volatile fatty acid production. Certain species within the *Prevotella* genus, such as *Prevotella bryantii*, may be linked to decreased live weight gain in cattle (Jami et al., 2013; Myer et al., 2017). This negative association may be due to the fermentation products generated by *Prevotella*. These products have the potential to alter nutrient utilization and energy metabolism in the host animal. Similarly, *Succiniclasticum*, a genus known for its involvement in succinate metabolism, fermenting succinate to propionate in the rumen, has been associated with decreased feed efficiency and reduced live weight gain in Holstein calves (Ortiz-Chura et al., 2021). *Succiniclasticum* species compete for substrates with other rumen microbes, potentially altering the efficient function of beneficial rumen fermentation pathways and affecting the overall nutrient availability and utilization by the host animal. Such a disruption in rumen fermentation could contribute to suboptimal growth performance of cattle. In contrast, the association of *Terrisporobacter* and *Carnobacterium* with live weight gain in cattle remains relatively underexplored in the literature. *Terrisporobacter*, a less-studied genus within the context of cattle rumen microbiota, lacks clear evidence regarding its specific impact on live weight gain. Similarly, although *Carnobacterium* has been identified in the rumen microbiota of cattle, its role in influencing host performance, including live weight gain, requires further investigation.

Some studies have documented positive associations between the presence of *Alloprevotella* and gut morphology which suggests potential benefits for nutrient absorption and animal performance (Jami et al., 2013; Myer et al., 2017). When considering the use of probiotics to modify the GIT microbiome, it is crucial to acknowledge the complexity of microbial interactions within the gastrointestinal tract. Specific bacterial taxa, such as *Alloprevotella*, may exert varied effects on gut morphology depending on a multitude of contributing factors, such as host physiology, ration composition, and environmental conditions. *Firmicutes*, a dominant phylum in the rumen microbiota, encompasses diverse genera involved in fiber degradation and VFA production, which are crucial for rumen fermentation and nutrient utilization (McCann et al., 2014; Henderson et al., 2015). Studies have shown that *Firmicutes* play essential roles in the breakdown of complex carbohydrates into short-chain or VFA, which are subsequently utilized as energy sources by the host animal (Wallace et al., 2014). The *Subdoligranulum* and *Butyricicoccus* genera within the *Firmicutes* phylum have been linked to increased butyrate production in the rumen. These bacterial genera contribute to butyrate synthesis through their fermentation of dietary fiber and other substrates and thereby indirectly influence rumen epithelial morphology and function (Paillard et al., 2007). *Ruminiclostridium9*, a member of the *Clostridia* class within *Firmicutes*, has been implicated in rumen fermentation processes and may play a role in shaping rumen villi morphology. Although specific studies directly linking *Ruminiclostridium9* to rumen development are limited, its presence in the rumen microbiota suggests potential interactions with host epithelial cells and involvement in rumen fermentation dynamics (Cunha et al., 2011).

Additional research is needed to elucidate the specific mechanisms by which members of the phylum *Firmicutes*, such as *Subdoligranulum, Butyricicoccus*, and *Ruminiclostridium9*, exert their influence on rumen morphology and function. This must include an investigation of any potential interactions with host physiology, ration composition, and environmental factors. Several studies emphasized the significance of *Prevotellaceae*, specifically *Prevotella7*, on GIT development and performance in cattle. *Prevotellaceae*, a family of bacteria abundant in the rumen microbiota, play a crucial role in fiber degradation, VFA production, and nutrient metabolism (Myer et al., 2015). *Prevotella7* abundance was reported to be positively correlated with rumen development and function in young ruminants (Yáñez-Ruiz et al., 2015; Roehe et al., 2016). Carbohydrate-active enzyme–producing bacterial genera like *Prevotella* and *Ruminiclostridium9* can optimize fiber digestion and potentially enhance growth outcomes (Flint et al., 2012; Huws et al., 2018). During the early stages of life, the establishment of *Prevotella7* populations in the rumen coincides with the maturation of the rumen epithelium and the development of rumen papillae, both of which are crucial for nutrient absorption and metabolism (Henderson et al., 2015). *Prevotella7* may contribute to these processes by facilitating the breakdown of dietary fibers and the production of fermentation by-products essential for rumen epithelial health and development. *Prevotella7* has also been implicated in modulating the immune response and inflammatory processes within the GIT, potentially influencing GIT morphology and function (Jami et al., 2014). Through interactions with the host immune system and modulation of the inflammatory process, *Prevotella7* may indirectly affect GIT development and integrity, ultimately affecting animal health and performance. Although *Prevotella*7 may play a role in rumen development and function, its direct influence on average weight gain or overall performance requires further investigation.

The correlations between the abundance of *Parabacteroides* and GIT development and weight gain in cattle have not been extensively studied. *Parabacteroides*, a genus within the *Bacteroidetes* phylum, is part of the complex microbial community residing in the cattle gut. Studies have reported that the gut microbiota composition, including the presence of *Parabacteroides*, can influence GIT development by modulating processes such as epithelial cell proliferation, immune system maturation, and nutrient metabolism (Kamada et al., 2013). *Odoribacter*, a member of the *Bacteroidetes* phylum, has been isolated in the gut microbiota of ruminants. *Prevotella2* and *Prevotella9*, also within the *Prevotella* genus prevalent in the rumen microbiota, are known for fiber degradation and volatile fatty acid production. These processes are critical for rumen fermentation and nutrient utilization by ruminants (Jami et al., 2013; Myer et al., 2015). *Treponema2*, belonging to the *Spirochaetes* phylum, has been identified in the rumen and feces of cattle and plays a role in fiber degradation and rumen fermentation (Fernando et al., 2010). Although all these bacterial genera are recognized components of the cattle gut microbiota, their precise roles in GIT development and weight gain necessitate further investigation. Studying the interactions between these bacterial taxa and host physiology may yield valuable insights into optimizing cattle health and productivity. Although certain gut microbiota compositions have been linked to variations in weight gain and feed efficiency in cattle, the specific contribution of *Parabacteroides* to these parameters is not well-defined (Myer et al., 2015, Li et al., 2019). *Odoribacter, Prevotella2, Prevotella9*, and *Treponema2* are important for fermentation and nutrient metabolism essential to rumen function. However, their exact roles in gastrointestinal development and weight gain are not yet fully understood.

This study investigates the correlation between GIT morphological changes, enteric microbiota composition, and changes in live weight in dairy calves. Preliminary insights from these correlations could enhance strategies for improving calf health and weight gain. In both the control and DFM-supplemented groups, 4 bacterial genera were identified as potential biomarkers associated with lower ADG. Additionally, 14 taxa that could be linked to gastrointestinal histopathology morphological measurements were identified across the experimental groups. *Odoribacter, Prevotella2, Prevotella9*, and *Treponema2* are crucial for fermentation and nutrient metabolism in the rumen, but their exact roles in gastrointestinal development and weight gain are not fully understood. Given the complex interactions within the gut microbiome, further research is needed to clarify their direct effects on cattle health and performance.
